# School grade and sex differences in domain-specific sedentary behaviors among Japanese elementary school children: a cross-sectional study

**DOI:** 10.1186/s12889-017-4221-z

**Published:** 2017-04-13

**Authors:** Kaori Ishii, Ai Shibata, Minoru Adachi, Yoshiyuki Mano, Koichiro Oka

**Affiliations:** 1 Faculty of Sport Sciences, Waseda University, 2-579-15 Mikajima Tokorozawa, Saitama, 359-1192 Japan; 2 Faculty of Health and Sport Sciences, University of Tsukuba, 3-29-1 Otsuka Bunkyo, Tokyo, 112-0012 Japan; 3 Graduate School of Education, Okayama University, 1-1-1 Tsushima-naka Kita, Okayama, 700-8530 Japan

**Keywords:** Domain, Sedentary behavior, Child, Self-report

## Abstract

**Background:**

It is vital to reduce the proportion of sedentary behavior in children. Understanding the duration and behavioral context is needed. The present study examined school-grade and sex differences in domain-specific sedentary times and concurrence with screen-time guidelines among Japanese elementary school children.

**Methods:**

A total of 625 children (330 boys) were surveyed in 2010 and 2014. Using a questionnaire, data regarding participants’ grade (first through third grades: lower grades; fourth through six grades: higher grades), sex, weight, and height were collected in addition to the time spent per day engaging in each specific sedentary behavior separately: (1) reading or listening to music, (2) TV or video viewing, (3) TV game use, (4) internet use excluding class, (5) homework, and (6) car travel. Two-way analysis of covariance and logistic regression analyses, adjusted for BMI and moderate to vigorous physical activity, were used to examine school-grade and sex differences in sedentary behaviors and the independent risk of exceeding recommended total daily screen time (< 2 h).

**Results:**

On 625 children, mean minutes (SD) of sedentary behavior per week in (1) – (6) were 90.3 (123.4), 535.0 (356.6), 167.3 (222.1), 23.9 (70.9), 264.9 (185.3), and 33.4 (61.2) in weekdays and 42.1 (70.0), 323.9 (232.0), 123.0 (96.4), 15.8 (49.9), 74.4 (96.4), and 71.3 (84.9) in weekends, respectively. There were differences in the minutes of sedentary behavior between participants of 2010 and 2014; e.g., TV game use and homework in weekdays and weekdays and car travel in weekends. Boys spent more time in TV game use, and girls spent more time reading, listening to music, doing homework, and car travel. Higher-grade students spent more time reading or listening to music, using a computer, and doing homework. Higher-grade students were 2.09 times (95% CI: 1.32 − 3.30) in whole week, 2.08 times (95% CI: 1.45 − 3.00) in weekday, and 1.88 times (95% CI: 1.29 − 2.74) in weekend more likely to spend ≥2 h per day in domains (2) − (4) (screen-time) than lower-grade students.

**Conclusions:**

Time spent engaging in each domain-specific sedentary behavior differed according to sex and school grade. Higher-grade students were less likely to meet screen-time guidelines. These findings highlight the need for domain-focused strategies to decrease sedentary behavior in Japanese school-age children.

## Background

Excessive sedentary behavior in childhood, such as habitual television (TV) viewing or video game play, is associated with weight gain/obesity [1], cardiometabolic disease risk [2], and poor mental health [3], independent of physical activity levels [4]. Despite these risks, the proportion of children who spend excessive time in sedentary behavior is increasing worldwide [4]. Reducing sedentary behavior among children is critical in order to stem the current increase in lifestyle-associated diseases (e.g., type-2 diabetes and hypertension). Current activity guidelines [5, 6] recommend no more than 2 h per day of recreational screen time (i.e., watching TV, DVDs, or videos, playing TV games, and computer use), and limiting sedentary transportation and reducing prolonged periods of sitting or time indoors [4]. However, the development of more effective intervention strategies requires a greater understanding of behavioral context, such as the places and social situations associated with inactivity, as well as the individual times spent on specific sedentary behaviors in target populations.

Numerous studies have examined sedentary behavior in school-age children, and many have focused on relationships to diet, obesity, diabetes, and cardiovascular disease [7–10] or sex differences [11, 12]. Others have examined domain-specific sedentary times, particularly time spent in TV viewing [13–17] or total screen time [18–23]. Such studies have revealed regional differences in domain-specific sedentary times. For instance, the proportion of children meeting the <2 h/day recreational screen time guideline differed between study populations in Norway [24] and Canada [25]. Other types of sedentary behaviors also vary by country [26], as these behaviors may be heavily dependent on culture, the socioeconomic status, and lifestyle.

While documenting behavior characteristics in target populations is critical for effective targeted interventions [27], few studies have comprehensively assessed differences in domain-specific sedentary behaviors and compliance with activity or screen-time guidelines between sexes and among different age groups in childhood. Descriptive epidemiological studies have suggested that differences in sedentary behavior between boys and girls are domain-specific [28–30], and that sedentary behavior increases with age [7, 31]. Therefore, the aim of this present study was to examine domain-specific sedentary behaviors and compliance with screen-time guidelines in Japanese school children from 6 to 11 years of age in order to develop targeted interventions according to age (or grade level) and sex.

## Methods

### Participants and data collection

The present stratified sampling cross-sectional study (*n* = 967) was conducted in 9 public elementary schools, 1 in Okayama in 2010 and 8 in Tokyo in 2014 agreed to participate in the present study. The children ranged in age from 6 − 11 years. In the Okayama school, students were enrolled from 3 first grade, 2 s grade, 3 third grade, 2 fourth grade, 3 fifth grade, and 3 sixth grade classes. All classes enrolled in the 8 Tokyo schools were fifth grade (19 in total, 5 schools with 2 classes, 3 schools with 3 classes). The present study included all students from these grades. Tokyo is an urban area while Okayama is a mid-sized regional suburban city. Permissions were granted by the Boards of Education of each city and prefecture, and by the principals of each school after research goals and methodology were fully explained. They were informed of study goals and importance, and assured that participant confidentially would be maintained. Children received a questionnaire from their teachers and were instructed to complete it at home in April in 2010 and September to October in 2014. The package contained a letter to parents/guardians explaining the ethical considerations of the study and requesting their participation. Returning the questionnaire constituted informed consent. Participation was voluntary, and the confidentiality of the participants was ensured.

A previous study [32] suggested that children younger than 10 years of age are unable to report their activity patterns accurately or reliably. Alternatively, parental reports of physical activity among 6 year olds has shown to have a strong correlation with heart rate measures during physical activity [33]. Therefore, parents or guardians of children in first through third grades (lower grades) were asked to complete the questionnaire with their child. Children in fourth grade and above (over 10 years old, higher grades) were asked to complete the questionnaire without parental or guardian assistance.

The study was approved by the human research ethics committee at Waseda University.

### Measures

#### Demographic and anthropometric variables

Data on participant school grade, sex, weight, and height were obtained from school records. Participants were divided into lower-grade (grades 1 − 3) or higher-grade (grades 4 − 6) groups. During normal school medical check-ups in April 2010 and 2014, weight and height were measured by a trained nurse or class teacher using the school’s stadiometers and weighing scales. Body mass index (BMI) was calculated from the height and weight data (weight/height^2^).

#### Domain-specific sedentary behavior and physical activity

Domain-specific sedentary behaviors were collected using a questionnaire. Sedentary behavior was divided into 6 domains [34]: (1) reading or listening to music, (2) TV or video viewing, (3) TV game use, (4) internet or e-mail (computer or tablet) use outside of class, (5) doing homework or assignments, and (6) car travel for transport. Participants were asked how many days on average per week and how much time (hours and minutes) on average per day they engaged in these sedentary behaviors during weekdays and weekends in each domain, and then the values were multiplied by the frequency per week and minutes per day. Each domain-specific sedentary behavior was examined separately, and we calculated the average total number of minutes for each school week (Monday − Friday) and weekend (days × minutes per day). The data were assessed on a scale currently used for a national survey of Japanese students [34]. Screen time was calculated using the total of domains (2), (3), and (4). For the screen-time cut-off point, the present study used the American Academy of Pediatrics guideline of a maximum of 2 h per day [5]. Physical activity was also measured through the questionnaire. The scale was originally developed to assess children’s physical activity by reference to a previous study [35]. Although it is widely agreed that children should have 60 min or more of physical activity daily, no precise assessment scale has been established in Japan. Therefore, participants were asked whether they engaged in moderate to vigorous physical activity (MVPA) that induces harder than normal breathing, such as brisk walking, cycling, dancing, or swimming, for at least 60 min per day, and, if so, how many days did they engage in MVPA in the past week.

### Statistical analyses

Two-way analysis of covariance, adjusted for BMI z-scores, number of days with MVPA for at least 60 min z-scores, and geographic area, was used to examine school-grade and sex differences in sedentary behaviors. Logistic regression analyses were conducted to examine independent relationships between risk of exceeding 2 h/day total screen-time and both sex and school grade, adjusted for BMI z-scores, number of days with MVPA for least 60 min z-scores, and geographic area. Participants with any missing data were excluded from the analysis. Missing data were defined as any questions that were unanswered. All statistical analyses were performed using PAWS Statistics 21 and results were considered significant at *p* < 0.05.

## Results

### Demographics, sedentary behaviors, and physical activity of the participants

A total of 625 Japanese children (330 boys and 295 girls, 322 in Okayama in 2010 and 303 in Tokyo in 2014) completed the survey (rate of participation for analysis: 64.6%, 342 participants were excluded). Table 1 shows the demographic characteristics of participants as well as the 6 sedentary domains assessed {time spent [mean minutes (SD) per week] in reading or listening to music, TV or video viewing, TV game use, computer or tablet use excluding class, doing homework or assignments, and car travel for transport] separately for weekdays and weekends and the physical activity of the study participants. The sedentary times for each of the six domains were 90.3 (123.4), 535.0 (356.6), 167.3 (222.1), 23.9 (70.9), 264.9 (185.3), and 33.4 (61.2) on weekdays, and 42.1 (70.0), 323.9 (232.0), 123.0 (96.4), 15.8 (49.9), 74.4 (96.4), and 71.3 (84.9) on weekends, respectively. On both weekdays and weekends, the majority of sedentary time was spent in TV viewing, followed by TV game play. The mean number of days the participants engaged in MVPA per week were 3.0 (SD = 2.2). The mean BMI was 16.9 kg/m^2^ (standard deviation [SD] = 2.7 kg/m^2^). There were differences in the BMI (t(623) = 3.49, *p* < 0.00), percentage of participants in lower grades/higher grades (χ^2^ = 225.1, *p* < 0.00), minutes of sedentary behavior; TV game use and homework in weekdays (t(623) = 3.07, *p* = 0.002, t(623) = 2.45, *p* = 0.015) and weekends (t(623) = 2.22, *p* = 0.027, t(623) = 2.96, *p* = 0.003) and car travel in weekends (t(623) = −3.09, *p* = 0.002), mean days of physical activity (t(623) = 4.03, *p* < 0.00), and z-score of days of physical activity (t(623) = 4.05, *p* < 0.00) between 2010 and 2014.

**Table 1:** Descriptive characteristics of the study population and times spent in sedentary behaviors

	Number	Percent
Overall	625	100.0
Okayama in 2010	303	48.5
Tokyo in 2014	322	51.5
Sex		
Male	330	52.8
Female	295	47.2
Grade level		
Lower grades	173	27.7
Higher grades	452	72.3
Body mass index (kg/m^2^)		
Mean ± SD	16.9 ± 2.7	
Z score (median, 25 to 75%ile)	−0.19, −0.65 to 0.40	
Sedentary time, min/week		
Weekdays	Mean ± SD	
Reading or listening to music	90.3 ± 123.4	
TV or video viewing	535.0 ± 356.6	
TV game use	167.3 ± 222.1	
Computer use excluding class	23.9 ± 70.9	
Doing homework or assignments	264.9 ± 185.3	
Car travel	33.4 ± 61.2	
Weekends	Mean ± SD	
Reading or listening to music	42.1 ± 70.0	
TV or video viewing	323.9 ± 232.0	
TV game use	123.0 ± 96.4	
Computer use excluding class	15.8 ± 49.9	
Doing homework or assignments	74.4 ± 96.4	
Car travel	71.3 ± 84.9	
Physical activity^a^, number of days/week		
Mean ± SD	3.0 ± 2.2	
Z score (median, 25 to 75 percent tile)	−0.12, −0.82 to 0.77	

*SD* standard deviation

^a^MVPA for at least 60 min

### Differences in domain-specific sedentary behavior according to sex and school grade

There were no significant main effects of sex and school grade on sedentary behavior, and a significant interaction between sex and school grade was found only for car travel (*P* = 0.019). However, girls spent significantly more time in the following sedentary behaviors: reading or listening to music on weekdays (*P* = 0.022), homework or assignments on weekdays (*P* = 0.013), and car travel on weekends (*P* = 0.019). Boys spent more time playing TV games than girls on both weekdays and weekends (both *P* < 0.001) (Table 2).

**Table 2:** Sex and school-grade differences in domain-specific sedentary behavior in Japanese children, 2010 and 2014

	Males				Females				Sex				School grade				Sex × school grade			
	Lower grade		Higher grade		Lower grade		Higher grade													
	mean	SE	mean	SE	mean	SE	mean	SE	F	df	η^2^	p	F	Df	η^2^	p	F	df	η^2^	p
Weekdays, min/week																				
Reading or listening to music	53.0	14.9	88.6	8.2	81.7	14.2	111.1	8.9	5.27	1618	0.008	0.022	5.58	1618	0.009	0.018	0.08	1618	0.000	0.781
TV or video viewing	505.3	43.2	520.1	23.9	495.8	41.3	582.6	25.9	0.67	1618	0.001	0.415	1.62	1618	0.003	0.204	1.27	1618	0.002	0.280
TV game use	196.3	25.8	232.9	14.3	98.6	24.7	106.6	15.4	33.45	1618	0.051	0.000	0.88	1618	0.001	0.350	0.56	1618	0.001	0.454
Computer use excluding class	2.5	8.4	33.9	4.7	5.5	8.1	35.4	5.0	0.01	1618	0.000	0.908	24.48	1618	0.038	0.000	0.13	1618	0.000	0.720
Doing homework or assignments	206.5	22.2	260.2	12.2	247.3	21.2	302.0	13.3	6.18	1618	0.010	0.013	7.00	1618	0.011	0.008	0.00	1618	0.000	0.978
Car travel	26.5	7.4	32.7	4.1	49.5	7.1	29.8	4.4	3.25	1618	0.005	0.072	0.99	1618	0.002	0.320	5.57	1618	0.009	0.019
Weekends, min/week																				
Reading or listening to music	26.7	8.4	40.3	4.7	35.1	8.1	53.7	5.1	2.97	1618	0.005	0.085	4.21	1618	0.007	0.041	0.16	1618	0.000	0.689
TV or video viewing	292.2	27.9	306.6	15.4	301.6	26.7	367.5	16.7	2.82	1618	0.005	0.094	2.41	1618	0.004	0.121	1.55	1618	0.003	0.213
TV game use	147.0	16.8	159.8	9.30	77.1	16.1	89.2	10.1	30.93	1618	0.048	0.000	0.64	1618	0.001	0.423	0.00	1618	0.000	0.976
Computer use excluding class	3.5	5.9	26.2	3.3	5.4	5.7	20.5	3.6	0.74	1618	0.001	0.391	25.6	1618	0.040	0.000	0.19	1618	0.000	0.666
Doing homework or assignments	56.8	11.5	73.1	6.4	51.6	11.0	93.3	6.9	0.75	1618	0.001	0.386	7.37	1618	0.012	0.007	2.22	1618	0.004	0.137
Car travel	68.5	10.2	60.5	5.6	86.1	9.7	78.8	6.1	5.55	1618	0.009	0.019	0.66	1618	0.001	0.417	0.00	1618	0.000	0.965

Adjusted for z-scores of BMI, number of days with moderate to vigorous physical activity (MVPA) for at least 60 min, and geographic area

*SE* standard error

Higher grades spent significantly more time than lower grades on reading or listening to music, in computer and tablet use, and on homework or assignments on weekdays and weekends (*P* < 0.001 for all).

### Association of non-attainment of screen-time guidelines with sex and school grades

Table 3 shows the odds ratios (ORs) of exceeding the screen-time guideline of 2 h per day. Higher-grade students (whole week, OR: 2.09, 95% confidence interval [CI]: 1.32–3.30; weekday, OR: 2.08, 95% CI: 1.45 − 3.00; and weekend, OR: 1.88, 95% CI: 1.29 − 2.74) were significantly more likely to spend >2 h per day of total screen time than lower-grade students. No significant relationship was observed for sex.

**Table 3:** Logistic regression analyses predicting screen time of >2 h/day

		% meeting guideline (≤ 2 h/day)	Whole week			Weekday			Weekend		
			OR	95% CI		OR	95% CI		OR	95% CI	
Sex	Male	35.5	1.21	0.87	1.69	1.19	0.86	1.65	1.17	0.82	1.68
	Female	40.7	1.00			1.00			1.00		
School grade	Higher grades	33.6	2.09	1.32	3.30	2.08	1.45	3.00	1.88	1.29	2.74
	Lower grades	49.1	1.00			1.00			1.00		

*OR* odds ratio, *CI* confidence interval

Adjusted for z-scores of BMI, number of days with MVPA of at least 60 min, and geographic area

## Discussion

The present study describes the weekly time spent in specific sedentary behaviors by Japanese elementary school children. Boys spent more time playing TV games, while girls spent more time reading or listening to music, doing homework or assignments, and car travel. Higher-grade students spent more time reading or listening to music, on computer or Internet use, and doing homework or assignments even after adjusted for the geographic areas or BMI. Moreover, approximately 60% of children spent time ≥ 2 h per day in sedentary behavior, the majority being devoted to screen time (TV watching and gaming). These findings indicate that programs for decreasing sedentary behavior in Japanese school children should be designed specifically for different age groups of boys and girls.

Sedentary behavior was high on both weekdays and weekends in the presented domains, particularly watching TV or videos and TV game use. Moreover, even compared with a previous study that reported a TV viewing time of 1.42 h (426 min/wk) on weekdays and 1.57 h (188.4 min/wk) on weekends among aged 5 years old [36], sedentary behavior was high. According to a national self-reported survey of 3210 children in 10 regions [37], Japanese children spend a substantial amount of time watching TV, playing video games, and using computers when home alone. Therefore, instructing children how to spend time when alone at home might be one effective strategy for decreasing sedentary behavior. However, the total average daily time spent on sedentary activity was even higher on weekends than weekdays. For instance, average TV viewing time was approximately more than 99 min daily on weekdays and 146 min daily on weekends. Alternatively, relatively little time was spent in computer use in this age group. Previous studies [38, 39] also found that leisure sedentary behavior time was higher on weekend days than weekdays. Therefore, family-focused weekend activities are a promising way to reduce sedentary behavior in children.

On weekdays, boys were more likely to spend time playing TV games and less likely to spend time reading, listening to music, and doing homework or assignments than girls. On weekends, boys were also more likely to spend time playing TV games and less likely to spend time in car travel than girls. These sex differences by weekday or weekend are consistent with findings from a previous cross-sectional study on a random sample of children aged 12.6-16.7 years from Scottish schools [39]. Several studies have reported that boys spend more time engaged in TV games than girls [12, 21, 23] and that girls spend more time engaged in reading and homework than boys [6, 10, 12]. Other studies have also reported greater TV video game play by boys than girls [18, 19] and more time spent reading by girls [9], although the differences were not statistically significant. As children’s leisure time activities clearly differ by sex [11], domain-focused approaches for reducing sedentary behavior must also be tailored specifically for boys and girls. Car travel on weekends frequently occurs with the family. According to a national self-reported survey of 3000 randomly sampled children [40], girls tend to spend more time on weekends with their families than do boys, which may explain the difference in car travel.

Higher grade students were more likely to spend time reading or listening to music, using a computer, and doing homework or assignments than were lower grade students. Other studies conducted on children aged 4-11.99 years have shown similar results in that higher-grade students spent more time on TV viewing (2.4 h/ day and 2.3 h/day in 9- to 11-year-old and 4- to 5-year-old boys, respectively, and 2.4 h/day and 2.2 h/day in 9- to 11-year-old and 4- to 5-year-old girls, respectively) and computer/video games (1.2 h/ day and 0.7 h/day in 4-5 years in 9- to 11-year-old and 4- to 5-year-old boys, respectively, and 0.8 h/day, 0.6 h/day in 9- to 11-year-old and 4- to 5-year-old girls, respectively) [18]. Increased time spent on computer use with advancing school grade or age is consistent across studies [7, 9, 17]. In contrast to numerous studies reporting that time spent on TV viewing increased with school grade or age [8, 13, 15, 16, 18], the present study found no significant differences. However, the average time spent watching TV in the present population (1.6 to 1.9 h per day on weekdays and 2.4 to 3.1 h per day on weekends) was low compared to previous estimates that ranged from 2.0 to 3.7 h per day in 35 countries and regions [14]. Nonetheless, TV viewing time still accounted by far the largest proportion of total sedentary time in Japanese children. No significant sex and grade differences were observed in the present study, which suggests that changing TV viewing habits should be a focus for reducing sedentary behavior in all Japanese children. Furthermore, sedentary behaviors in childhood are maintained in adolescence and into adulthood [22]; therefore, targeted strategies to reduce excessive screen time in childhood are crucial for encouraging lasting behavioral changes.

A national self-reported survey of 53,458 randomly selected Japanese children found that the proportion of time spent in learning activities in sedentary behavior outside of school hours without family increased with school grade [41]. Another self-reported survey of 3210 children from 10 areas of Japan found that lower-grade students spent more time with families on weekends than did higher-grade students [37], suggesting that lower-grade students received more social support from family than higher-grade students. Therefore, the present study showed that lower-grade students engaged in less sedentary behavior than higher-grade students and that family support is vital for reducing these sedentary behaviors, because they frequently occur at the family’s discretion, particularly in younger children.

In the present study, only 35.5% of boys and 40.7% of girls met the recommendation that children spend less than 2 h on screen time daily. This trend is comparable to that found in a study from China that asked about time spent watching videos, CDs, and DVDs; playing video games; and using a computer (46.7% and 42.3% of boys and girls, respectively) [42], study from the United States that asked about time spent watching TV and playing video games (26.7% and 35.0% of boys and girls, respectively) [19], and study from Canada that asked about the time spent watching TV and using a computer or playing video games (38.1% and 56.0% of boys and girls, respectively) [43]. Even after adjusted for z-scores of BMI, number of days with moderate to vigorous physical activity (MVPA) for at least 60 min, and geographic area, compared to lower-grade students, higher-grade students in our survey were 2.09 times during the whole week, 2.08 times on the weekday, and 1.88 times on the weekend more likely to spend ≥2 h/day on screen time. A study of Canadian children were asked to report the number of hours per day during the week that they spend watching TV or movies or playing video or computer games; in grades 5 − 8, the authors found that boys were 2.2 times more likely than girls to spend ≥3 h/day on screen time [25]. While the measuring methods of screen time differed among studies, making direct comparison difficult, the proportion of children who attained the 2-h limit was low in the present study group, especially for the higher-grade students. Effective strategies to decrease screen time are needed, especially in higher-grade students, including family-based interventions and removing TVs from children’s bedrooms [44, 45]. Further studies are required to identify factors that predict meeting or exceeding these guidelines.

The present study has some limitations. First, the study relied on self-reported measures, which have the potential for error due to different interpretations of the questions. However, objective measures such as accelerometers are free of behavioral context [46]. In addition, the present study did not gather data regarding all the domains of the children’s sedentary behavior, such as school time sitting, to give a more comprehensive overview of total sedentary time. Furthermore, the reliability and validity of the scales used in this study are unknown as it is not reported in previous studies [34]. However, it was used in a national survey of Japanese elementary school children [34]. In a previous study [34], parents/guardians of children in the first through third grades (6 − 8 years) were asked to complete the questionnaire after consulting with their children. Children in the fourth grade and above (9 − 11 years) were asked to complete the questionnaire without parental/guardian assistance to ensure reliability and validity. Although the present study used a previous representative survey of Japanese school children [34], the self-reported questionnaire was likely to be underappreciated. Second, data from lower-grade school children were collected with the assistance of a parent or guardian, whereas higher-grade children completed the questionnaire independently, which present two distinct sources of bias. Third, the questionnaire asked about each domain-specific sedentary behavior separately. However, when responding about domain-specific sedentary behaviors, the participants may not have reported on only one domain, e.g., they could have reported the time spent using a reading or listening to music while watching TV or video. Thus, it is necessary to consider that the self-reported sedentary time is overestimated. Fourth, there were differences in the grade level, BMI, and sedentary time between participants from Okayama in 2010 and Tokyo in 2014. Moreover, the difference in the period of measurements in 2010 and 2014 may have biased the data. Moreover, it is likely that Japanese children who are largely sedentary also have parents/guardians who are largely sedentary. However, the present study did not include items on parent’s/guardian’s sedentary behavior to adjust for these effects. Despite these limitations, the present study consider that the present study is meaningful because it is the first study to highlight sex and grade differences in domain-specific sedentary behaviors and attainment of screen-time guidelines in Japanese children. The present results provide an important foundation for the development of interventions to decrease sedentary behavior in Japanese children.

## Conclusions

Domain-specific sedentary behaviors differed according to sex and school grade. Higher-grade students were more likely to spend ≥2 h per day on screen time than lower-grade students. These findings highlight the need for strategies targeting specific domains in order to more effectively decrease sedentary behaviors in Japanese school-age children.

## Abbreviations

%: Percentage
BMI: Body mass index
CI: Confidence interval
kg: Kilogram
m: Meter
min: Minutes
MVPA: Moderate to vigorous physical activity
n: Number
OR: Odds ratio
SD: Standard deviation
SE: Standard error
TV: Television
